# The Presence of Both Tumor Spread through Air Spaces and Visceral Pleural Invasion May Increase Tumor Recurrence Risk in Non-Small Cell Lung Cancer

**DOI:** 10.5761/atcs.oa.25-00100

**Published:** 2025-10-10

**Authors:** Joshua R. Brady, Brittany Walker, Jocelyn C. Zajac, Daniel P. McCarthy, James D. Maloney, Malcolm M. DeCamp, Andrea L. Axtell

**Affiliations:** 1 Department of Surgery, University of Wisconsin Hospitals and Clinics, Madison, WI, USA; 2 Division of Cardiothoracic Surgery, University of Wisconsin School of Medicine and Public Health, Madison, WI, USA

**Keywords:** carcinoma, non-small cell lung, lung neoplasms, adenocarcinoma of lung

## Abstract

**Purpose:**

Tumor spread through air spaces (STAS) and visceral pleural invasion (VPI) are negative prognostic factors in lung cancer. However, it is unknown whether they have a compounding prognostic effect. Therefore, we analyzed the association between STAS and VPI with overall survival and recurrence.

**Methods:**

A retrospective cohort analysis was conducted on 421 adult patients who underwent pulmonary resection for non-small cell lung cancer at an academic institution between 2018 and 2022. Baseline characteristics were compared between patients who had STAS only, VPI only, or both STAS and VPI. Overall survival and cumulative recurrence were compared using the Kaplan–Meier method.

**Results:**

Of the 421 patients who underwent a pulmonary resection, 34 (8%) had both STAS and VPI. Their combined presence was associated with increased smoking pack-years, increased tumor size, and an increased presence of lymphovascular invasion. There was no overall survival difference (p = 0.190) between patients with both STAS and VPI and those with only one feature or neither. However, cumulative incidence of recurrence was increased (p = 0.001) for patients with both.

**Conclusion:**

The presence of STAS and VPI was not associated with decreased overall survival; however, their combined presence may have a compounding effect on recurrence risk.

## Introduction

Visceral pleural invasion (VPI) is defined as the presence of tumor cells beyond the elastic layer of the visceral pleura.^1)^ Since first being recognized in 1958 by Brewer et al.^2)^ as a poor prognostic factor, further research has confirmed that the presence of VPI in non-small cell lung cancer (NSCLC) is a high-risk feature, correlated with disease aggressiveness and associated with decreased overall survival and increased nodal involvement.^3,4)^ Due to significant research demonstrating that VPI is an independent risk factor for disease aggressiveness, the American Joint Committee on Cancer (AJCC) included it as a T-descriptor, with the current 8th edition TNM staging guideline for NSCLC upstaging tumors ≤3 cm from T1 to T2 if VPI is present.^5)^ VPI currently requires a pathologic assessment of tumor tissue for classification, with ongoing research focused on improving the predictive ability of preoperative imaging and other modalities for diagnosis, including autofluorescence bronchoscopy.^6,7)^

In comparison to VPI, tumor spread through air spaces (STAS) is a more recently recognized mechanism of invasion. STAS is defined as the presence of tumor cells in air spaces within the lung parenchyma beyond the tumor margin.^8)^ Recent studies support that STAS is an *in vivo* phenomenon rather than an artifact from specimen processing.^9)^ STAS has been associated with increased recurrence risk, particularly distant and locoregional recurrence in Stage I lung adenocarcinoma undergoing a limited resection, and has also been demonstrated to be an independent risk factor in disease recurrence, particularly locoregional recurrence.^10,11)^ Additional studies in adenocarcinoma, squamous cell carcinoma, and other types of lung cancer further demonstrate that STAS is a poor prognostic factor with associated effects on recurrence and survival.^12–15)^

Thus, while it is currently accepted that both tumor VPI and STAS are negative prognostic factors in NSCLC, it is unknown whether they have a compounding effect on prognosis. We therefore aimed to analyze a cohort of patients with NSCLC who underwent curative-intent surgical resection to evaluate whether the presence of both STAS and VPI on final pathology has a compounding effect on prognosis, to better inform possible additional post-resection treatments.

## Materials and Methods

### Patients and data collection

A retrospective cohort analysis using an institutional database was conducted on adult patients who underwent a 1st-time pulmonary resection for NSCLC between January 2018 and December 2022 at the University of Wisconsin Hospitals and Clinics. Patients prior to 2018 were excluded due to institutional non-reporting of STAS. All malignant histological types (adenocarcinoma, squamous cell carcinoma, and large cell carcinoma) and Stages IA–IIIB of disease were included for analysis. Disease staging was conducted according to the 8th edition of the AJCC TNM Staging Manual. Patients who underwent emergency resection and those with missing data for the presence or absence of either STAS or VPI were excluded. Institutional review board approval was obtained for this study (IRB# 2024-0754).

### Study design

The primary outcomes of this study were overall survival and disease recurrence. Overall survival was defined as the time from surgical resection to death from any cause. Recurrence was defined as evidence of tumor following complete resection. Recurrence included local (tumor in the same lobe or at the surgical margin of the original tumor), regional (tumor in a second ipsilateral lobe or in the ipsilateral hilar or mediastinal lymph nodes), and distant (tumor in the contralateral lung, contralateral or supraclavicular lymph nodes, or outside the hemithorax). For all analyses, patients were censored at the date of their last known contact at our institution.

### Statistical analysis

Baseline clinical, operative, and pathologic characteristics were compared between patients with and without either STAS and/or VPI using an analysis of variance test for continuous variables and a chi-squared test for categorical variables. All data are presented as n (%) for categorical variables and as mean ± standard deviation for continuous variables. Overall survival for patients with and without either STAS and/or VPI was determined using the Kaplan–Meier method, followed by a log-rank test for differences between groups. Cumulative incidence of recurrence was analyzed using a competing risks method, accounting for death without recurrence as a competing event.

Within the overall cohort, a subgroup analysis was also conducted in patients with Stage IA disease with tumors less than or equal to 2.0 cm, for which overall survival and cumulative incidence of recurrence were compared. All analyses were completed using STATA v17.0 (STATA Corp., College Station, TX, USA). A p-value of less than 0.05 was considered statistically significant.

## Results

### Demographic data

Of 421 patients who underwent a pulmonary resection for Stage IA–IIIB NSCLC, 63 (15%) had STAS only, 99 (24%) had VPI only, and 34 (8%) had both STAS and VPI on final pathologic assessment. Baseline clinical characteristics for the entire patient cohort and the subgroups of patients with either STAS, VPI, both, or neither are presented in **Table 1**. There was no difference between the 4 subgroups (STAS only, VPI only, both STAS and VPI, or neither STAS nor VPI) based on age or gender. However, patients with both STAS and VPI were more likely to have a higher pack-year smoking history (57 vs. 41 years [VPI only] vs. 41 years [STAS only] vs. 41 years [neither STAS or VPI], p = 0.016) and COPD (62% vs. 39% [VPI only] vs. 29% [STAS only] vs. 37% [neither STAS nor VPI], p = 0.008). Clinicopathologic features and operative characteristics for the entire patient cohort and the subgroups of patients with either STAS, VPI, both, or neither feature are presented in **Table 2**. There were no significant differences among the 4 subgroups regarding overall histological type. However, there were significant differences among the subgroups regarding predominant histologic subtype (p ≤0.001) and grade, with tumors that had both STAS and VPI more likely to be poorly differentiated (74% vs. 65% [VPI only] vs. 50% [STAS only] vs. 38% [neither STAS nor VPI], p ≤0.001). Additionally, the presence of both STAS and VPI was associated with increased tumor size (3.3 vs. 2.8 cm [VPI only] vs. 2.3 cm [STAS only] vs. 2.0 cm [neither STAS nor VPI], p ≤0.001) and concurrent lymphovascular invasion (LVI) (65% vs. 53% [VPI only] vs. 37% [STAS only] vs. 22% [neither STAS nor VPI], p ≤ 0.001). Furthermore, the presence of both STAS and VPI was associated with more advanced pathologic stage (p ≤0.001), with a majority of patients with only STAS (73%), only VPI (62%), or neither feature (81%) having Stage I disease, while a majority of patients with both STAS and VPI (62%) had Stage IIA disease or higher, with 44% having Stage IIB disease.

**Table 1:** Baseline clinical characteristics

Characteristic	Overall (n = 421)	No STAS or VPI (n = 225)	STAS+ only (n = 63)	VPI+ only (n = 99)	STAS+ and VPI+ (n = 34)	p-Value
Age (years)	68 ± 9	68 ± 9	66 ± 9	69 ± 9	68 ± 11	0.408
Female gender	245 (58%)	140 (62%)	37 (59%)	48 (48%)	20 (59%)	0.148
Race						0.676
Caucasian	404 (96%)	216 (96%)	58 (92%)	96 (97%)	34 (100%)	
Black	11 (3%)	6 (3%)	3 (5%)	2 (2%)	0 (0%)	
Hispanic	11 (3%)	5 (2%)	2 (3%)	1 (1%)	2 (6%)	
Asian	5 (1%)	2 (1%)	2 (3%)	1 (1%)	0 (0%)	
Unknown/other	2 (0%)	2 (1%)	0 (0%)	0 (0%)	0 (0%)	
BMI >30	129 (31%)	73 (32%)	26 (41%)	19 (19%)	11 (29%)	**0.020**
Smoking history						0.191
Never	76 (18%)	37 (16%)	17 (27%)	17 (17%)	5 (14%)	
Current	112 (27%)	59 (26%)	10 (16%)	33 (33%)	10 (29%)	
Former	233 (55%)	129 (57%)	36 (57%)	49 (50%)	19 (56%)	
Pack-year history	41 ± 26	39 ± 24	41 ± 23	41 ± 26	57 ± 42	**0.016**
Hypertension	280 (67%)	147 (65%)	47 (75%)	66 (67%)	20 (59%)	0.379
Coronary artery disease	83 (20%)	46 (20%)	9 (14%)	19 (19%)	9 (26%)	0.461
Congestive heart failure	8 (2%)	4 (2%)	3 (5%)	1 (1%)	0 (0%)	0.276
Cerebrovascular disease	47 (11%)	22 (10%)	12 (19%)	10 (10%)	3 (9%)	0.196
Diabetes mellitus	71 (17%)	36 (16%)	10 (16%)	16 (16%)	9 (26%)	0.487
Pulmonary hypertension	18 (4%)	12 (5%)	3 (5%)	3 (3%)	0 (0%)	0.467
COPD	155 (37%)	77 (34%)	18 (29%)	39 (39%)	21 (62%)	**0.008**
Interstitial fibrosis	9 (2%)	2 (1%)	2 (3%)	4 (4%)	1 (3%)	0.282
Prior cardiothoracic surgery	34 (8%)	19 (8%)	4 (6%)	6 (6%)	5 (15%)	0.416
Pulmonary function tests						
FEV1 (predicted %)	88 ± 19	88 ± 18	92% ± 17	86% ± 22	87% ± 18	0.232
DLCO (predicted %)	77 ± 20	77 ± 20	86% ± 17	74% ± 20	78% ± 18	**0.005**

Bold values are significant as they are <0.05.

STAS: tumor spread through air spaces; VPI: visceral pleural invasion; BMI: body mass index; COPD: chronic obstructive pulmonary disease; FEV1: forced expiratory volume in 1 second; DLCO: diffusing capacity for carbon monoxide

**Table 2:** Clinicopathologic features and operative characteristics

Characteristic	Overall (n = 421)	No STAS or VPI (n = XX)	STAS+ only (n = 63)	VPI+ only (n = 99)	STAS+ and VPI+ (n = 34)	p-Value
Histology						0.283
Adenocarcinoma	342 (81%)	172 (76%)	58 (92%)	82 (83%)	30 (88%)	
Squamous cell carcinoma	72 (17%)	47 (21%)	5 (8%)	16 (16%)	4 (12%)	
Large cell carcinoma	3 (1%)	3 (1%)	0 (0%)	0 (0%)	0 (0%)	
Other	3 (1%)	2 (1%)	0 (0%)	1 (1%)	0 (0%)	
Predominant histologic subtype						**<0.001**
Lepidic	37 (9%)	31 (19%)	4 (7%)	2 (2%)	0 (0%)	
Acinar	205 (49%)	101 (62%)	32 (56%)	54 (66%)	18 (60%)	
Papillary	15 (4%)	7 (4%)	6 (11%)	0 (0%)	2 (7%)	
Solid	37 (9%)	16 (10%)	5 (9%)	11 (13%)	5 (17%)	
Micropapillary	7 (2%)	3 (2%)	3 (5%)	0 (0%)	1 (3%)	
Mucinous	32 (8%)	6 (4%)	7 (12%)	15 (18%)	4 (13%)	
Grade						**<0.001**
Well differentiated	42 (10%)	37 (17%)	4 (6%)	1 (1%)	0 (0%)	
Moderately differentiated	170 (40%)	100 (45%)	27 (44%)	34 (34%)	9 (26%)	
Poorly differentiated	204 (48%)	84 (38%)	31 (50%)	64 (65%)	25 (74%)	
Tumor size (cm)	2.3 ± 1.5	2.0 ± 1.2	2.3 ± 1.6	2.8 ± 1.6	3.3 ± 2.3	**<0.001**
Margin distance (cm)	2.7 ± 1.8	2.8 ± 1.9	2.7 ± 1.8	2.5 ± 1.9	2.8 ± 1.5	0.653
Lymphovascular invasion	146 (35%)	50 (22%)	23 (37%)	51 (53%)	22 (65%)	**<0.001**
Pathologic stage						**<0.001**
Stage I	302 (72%)	182 (81%)	46 (73%)	61 (62%)	13 (38%)	
Stage IIA	11 (3%)	5 (2%)	1 (2%)	5 (5%)	1 (3%)	
Stage IIB	69 (16%)	26 (12%)	9 (14%)	19 (19%)	15 (44%)	
Stage IIIA	39 (7%)	8 (4%)	7 (11%)	10 (10%)	4 (12%)	
Stage IIIB	5 (1%)	1 (0%)	0 (0%)	4 (4%)	1 (3%)	
PDL-1 status						0.107
<1%	72 (17%)	41 (18%)	12 (19%)	10 (10%)	9 (26%)	
1%–50%	106 (25%)	45 (20%)	18 (29%)	33 (33%)	10 (29%)	
>50%	34 (8%)	17 (8%)	5 (8%)	8 (8%)	4 (12%)	
Unknown	209 (50%)	122 (54%)	28 (44%)	48 (48%)	11 (32%)	
Neoadjuvant therapy	15 (4%)	8 (4%)	2 (3%)	4 (4%)	1 (3%)	0.988
Operation						0.688
Wedge resection	29 (7%)	16 (7%)	5 (8%)	7 (7%)	1 (3%)	
Segmentectomy	74 (18%)	45 (20%)	8 (13%)	17 (17%)	4 (12%)	
Lobectomy	318 (76%)	164 (73%)	50 (79%)	75 (76%)	29 (85%)	

Bold values are significant as they are <0.05.

STAS: tumor spread through air spaces; VPI: visceral pleural invasion; PD-L1: programmed death ligand 1

### Survival outcomes

**Fig. 1:**
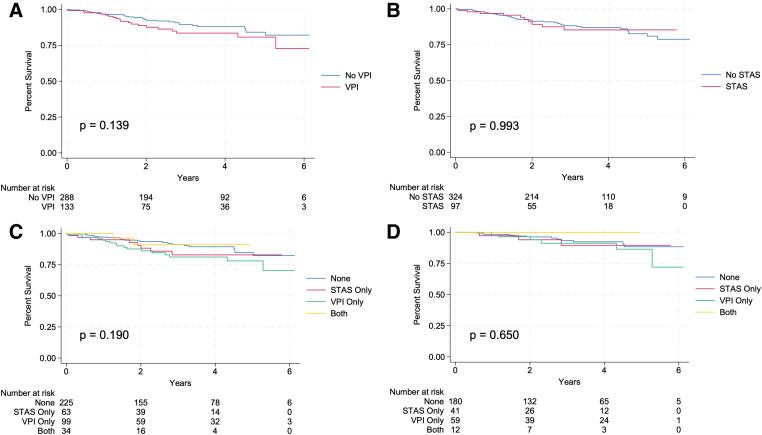
Overall survival. (**A**) Entire cohort stratified by the presence or absence of VPI. (**B**) Entire cohort stratified by the presence or absence of STAS. (**C**) Entire cohort stratified by the presence of only STAS, only VPI, neither, or both features. (**D**) Stage I disease stratified by the presence of only STAS, only VPI, neither, or both features. STAS: tumor spread through air spaces; VPI: visceral pleural invasion.

There was no difference in overall survival within the entire cohort of patients when stratified by the presence of VPI (p = 0.139; **Fig. 1A**) or by the presence of STAS (p = 0.993; **Fig. 1B**). Furthermore, there was no difference in overall survival within the entire cohort of patients with STAS only, VPI only, both STAS and VPI, or neither on final pathology (p = 0.190; **Fig. 1C**). Within the Stage I (tumors ≤2 cm) subgroup of patients with either one of these features, both, or none, this trend continued, with no significant difference in overall survival among these groups (p = 0.650; **Fig. 1D**).

### Recurrence outcomes

**Fig. 2:**
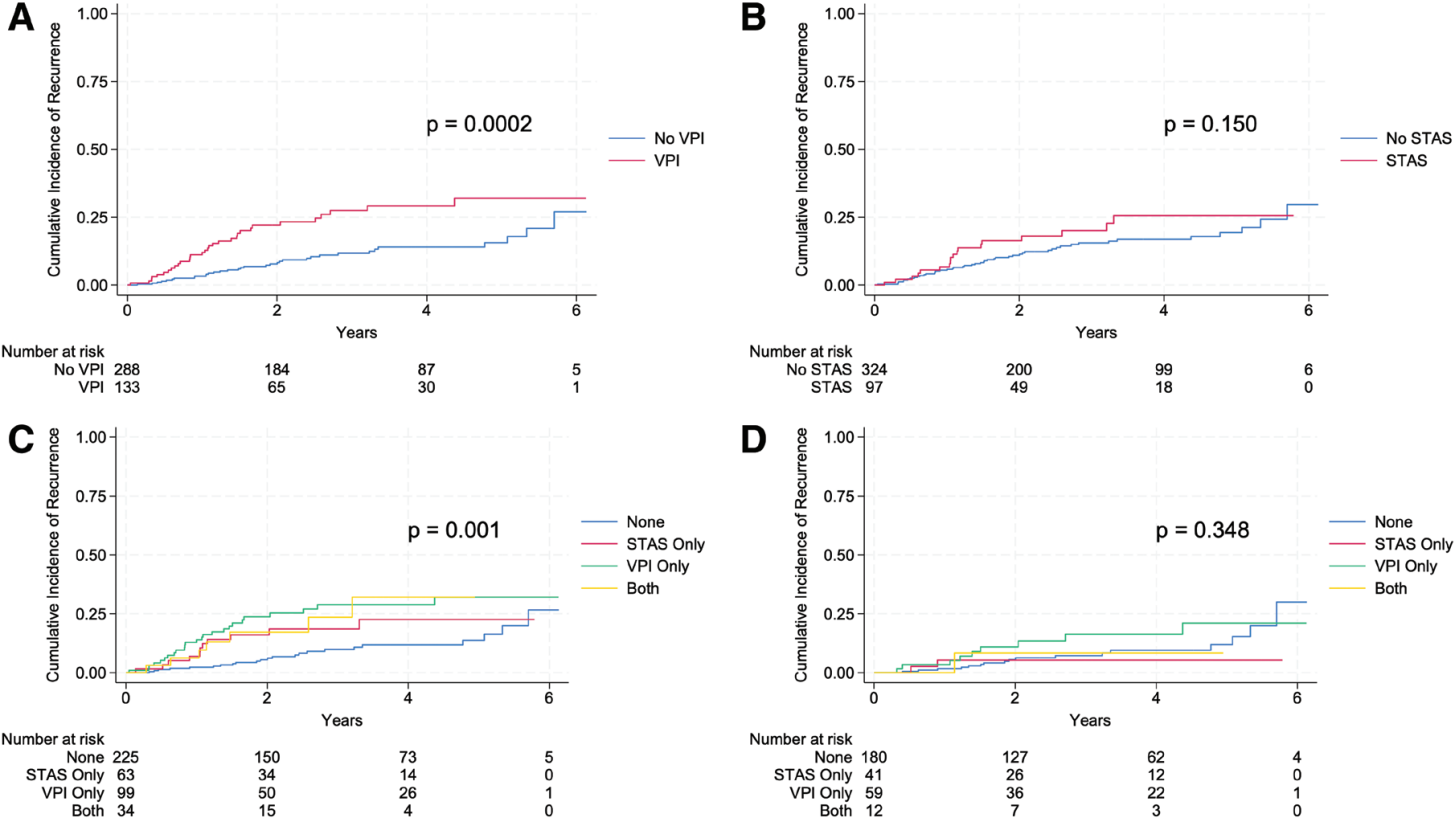
Cumulative incidence of recurrence. (**A**) Entire cohort stratified by the presence or absence of VPI. (**B**) Entire cohort stratified by the presence or absence of STAS. (**C**) Entire cohort stratified by the presence of only STAS, only VPI, neither, or both features. (**D**) Stage I disease stratified by the presence of only STAS, only VPI, neither, or both features. STAS: tumor spread through air spaces; VPI: visceral pleural invasion.

When the entire cohort of patients was stratified by VPI, the presence of VPI was associated with increased cumulative recurrence (p = 0.0002; **Fig. 2A**). In contrast, there was no significant association between STAS and cumulative recurrence within the entire cohort, although there was a trend toward increased recurrence (p = 0.150; **Fig. 2B**). However, there was a significant difference in the cumulative incidence of recurrence within the entire cohort of patients with STAS only, VPI only, both STAS and VPI, or neither on final pathology (p = 0.001; **Fig. 2C**). Among these groups, patients with VPI only and patients with both STAS and VPI had the highest cumulative incidence of recurrence (**Fig. 2C**). Of note, at 4 years after resection, patients with both STAS and VPI had the highest cumulative incidence of recurrence (**Fig. 2C**). Within the Stage I (tumors ≤2 cm) subgroup of patients with either one of these features, both, or none, there was no statistical difference in cumulative incidence of recurrence (p = 0.348; **Fig. 2D**); however, this was limited by a small sample size. Regarding recurrence frequency in the entire cohort, there was an overall 16% recurrence occurrence, with 10% of patients with neither STAS nor VPI, 17% of patients with STAS only, 25% of patients with VPI only, and 21% of patients with both STAS and VPI experiencing recurrent disease (**Table 3**). Within the entire cohort, there was a significant difference in recurrence location, with patients with both STAS and VPI more likely to have regional recurrence (57% vs. 16% [VPI only] vs. 18% [STAS only] vs. 21% [neither STAS nor VPI], p = 0.002) (**Table 3**). In contrast, patients with either STAS only or VPI only were more likely to have metastatic or distant recurrence (55% STAS only, 48% VPI only) (**Table 3**).

**Table 3:** Recurrence patterns

	Overall (n = 421)	No STAS or VPI (n = 225)	STAS+ only (n = 63)	VPI+ only (n = 99)	STAS+ and VPI+ (n = 34)	p-Value
Recurrence	66 (16%)	23 (10%)	11 (17%)	25 (25%)	7 (21%)	0.002
Local	26 (39%)	13 (57%)	3 (27%)	9 (36%)	1 (14%)	
Regional	14 (21%)	4 (17%)	2 (18%)	4 (16%)	4 (57%)	
Distant/metastatic	26 (39%)	6 (26%)	6 (55%)	12 (48%)	2 (29%)	

STAS: tumor spread through air spaces; VPI: visceral pleural invasion

## Discussion

In this institutional analysis of surgically resected Stage IA–IIIB NSCLC, we identified a 23% overall prevalence of STAS, a 32% overall prevalence of VPI, and an 8% occurrence of concurrent tumor STAS and VPI. We demonstrated that the presence of concurrent STAS and VPI is associated with increased smoking pack-years, increased prevalence of chronic obstructive pulmonary disease (COPD), concurrent LVI, poorly differentiated tumor grade, and larger tumor size. In the entire cohort of patients with Stage IA–IIIB disease, the presence of both STAS and VPI was not predictive of a difference in overall survival. Interestingly, in the entire cohort of patients with Stage IA–IIIB disease, there was a significant difference among all groups, with patients with VPI only and patients with both STAS and VPI having the highest cumulative incidence of recurrence. Notably, patients with both STAS and VPI had the highest cumulative recurrence at 4 years after resection. Furthermore, patients with both STAS and VPI were more likely to have recurrence detected at the regional level, compared to distant recurrence for patients with only STAS or only VPI.

In the overall cohort of patients, VPI was associated with significantly increased overall recurrence and a trend toward decreased overall survival that did not reach statistical significance, likely due to the small sample size. These results align with VPI’s role as a T-descriptor in the current 8th edition AJCC TNM staging guidelines and with prior studies demonstrating that VPI is a poor prognostic factor in NSCLC.^3–5)^ For example, a retrospective cohort study of 886 patients between January 2000 and December 2007 found VPI to be a significant independent prognostic factor by multivariate analysis in tumors ≤7 cm (p = 0.0002), with VPI-positive tumors ≤3 cm having an increased risk of lymph node metastasis (p = 0.0003).^16)^

In the overall cohort of patients, STAS was associated with a trend toward increased cumulative recurrence, particularly during the first 4 years following resection. Furthermore, in the overall patient cohort, STAS was not associated with a difference in overall survival. This differs from previous studies that have demonstrated STAS to have a significant negative effect on survival and an association with increased cumulative recurrence, particularly at the locoregional level.^10,11)^ Furthermore, a recent meta-analysis of 3754 patients over 14 studies demonstrated that STAS was associated with a pooled hazard ratio of 1.975 for recurrence-free survival (95% confidence interval [CI]: 1.691–2.307, p ≤0.001) and a pooled hazard ratio of 1.75 for overall survival (95% CI: 1.375–2.227, p ≤0.001).^17)^ Differences between the results of our study and the current literature regarding VPI and STAS likely stem from the small sample size and censoring in the setting of loss to follow-up. Notably, the number of patients experiencing recurrent disease was 11 in the STAS-only subgroup, 25 in the VPI-only subgroup, and 7 in the STAS and VPI subgroup, which, combined with censoring, may make this study underpowered to detect a difference in the cumulative incidence of recurrence.

This study has limitations within which the findings must be interpreted. First, this study is a cohort study from a single institution in which the predominant patient population identifies as White, potentially limiting the findings’ generalizability to larger, more diverse populations. Second, as discussed previously, the cohort size of this study was small, particularly the subgroup of patients with both tumor STAS and VPI on final pathologic assessment (n = 34). This is in part secondary to institutional non-reporting of tumor STAS prior to 2018. The small sample size also limits the generalizability of the study’s conclusions and limited our ability to perform a multivariate analysis to potentially delineate the effects of other high-risk features (increased smoking pack-years, concurrent LVI, larger tumor size, etc.) with which concurrent STAS and VPI were also associated. Furthermore, the cumulative incidence of recurrence may be underrepresented due to limitations in documentation regarding patients who received their cancer care outside of our health system, as these patients were censored at the time of their last known contact at our institution.

## Conclusion

In conclusion, concurrent tumor STAS and VPI is associated with other high-risk features, including increased smoking pack-years, COPD, concurrent LVI, poorly differentiated tumor grade, and larger tumor size. While concurrent tumor STAS and VPI had no significant effect on overall survival, there may be a trend toward increased cumulative incidence of recurrence, particularly recurrence detected at the regional level (tumor in a second ipsilateral lobe or in the ipsilateral hilar or mediastinal lymph nodes). Future research is needed to evaluate the implications of having both features present on final pathologic assessment as it relates to prognosis and potential additional post-resection treatments.

## Declarations

### Data availability

All data are included in this article. Further inquiries can be directed to the corresponding author.
